# Combining WGCNA and machine learning to construct basement membrane-related gene index helps to predict the prognosis and tumor microenvironment of HCC patients and verifies the carcinogenesis of key gene CTSA

**DOI:** 10.3389/fimmu.2023.1185916

**Published:** 2023-05-23

**Authors:** Weijie Sun, Jue Wang, Zhiqiang Wang, Ming Xu, Quanjun Lin, Peng Sun, Yihang Yuan

**Affiliations:** ^1^ Department of General Surgery, Tongren Hospital, Shanghai Jiao Tong University School of Medicine, Shanghai, China; ^2^ Department of Infectious Diseases, The First Affiliated Hospital of Anhui Medical University, Hefei, China

**Keywords:** hepatocellular carcinoma, basement membranes, prognosis, immunotherapy, machine learning, ScRNA-seq, CTSA, *vitro* experiment

## Abstract

Hepatocellular carcinoma (HCC) is a malignant tumor with high recurrence and metastasis rates and poor prognosis. Basement membrane is a ubiquitous extracellular matrix and is a key physical factor in cancer metastasis. Therefore, basement membrane-related genes may be new targets for the diagnosis and treatment of HCC. We systematically analyzed the expression pattern and prognostic value of basement membrane-related genes in HCC using the TCGA-HCC dataset, and constructed a new BMRGI based on WGCNA and machine learning. We used the HCC single-cell RNA-sequencing data in GSE146115 to describe the single-cell map of HCC, analyzed the interaction between different cell types, and explored the expression of model genes in different cell types. BMRGI can accurately predict the prognosis of HCC patients and was validated in the ICGC cohort. In addition, we also explored the underlying molecular mechanisms and tumor immune infiltration in different BMRGI subgroups, and confirmed the differences in response to immunotherapy in different BMRGI subgroups based on the TIDE algorithm. Then, we assessed the sensitivity of HCC patients to common drugs. In conclusion, our study provides a theoretical basis for the selection of immunotherapy and sensitive drugs in HCC patients. Finally, we also considered CTSA as the most critical basement membrane-related gene affecting HCC progression. *In vitro* experiments showed that the proliferation, migration and invasion abilities of HCC cells were significantly impaired when CTSA was knocked down.

## 1 Introduction

Hepatocellular carcinoma (HCC) is responsible for about 90% of primary liver cancers (1). It is also one of the most fatal malignant tumors worldwide, with high morbidity and mortality rates (2, 3). The frequent occurrence of metastasis and recurrence is a major contributing factor to the poor prognosis of HCC patients (4). Despite the development of numerous drug combination strategies for the treatment of HCC, the current level of patient survival time has not yet met satisfactory standards. Consequently, there is an urgent need to identify new biomarkers that can more accurately predict the prognosis of HCC.

Basement membranes (BM) are a ubiquitous and unique type of extracellular matrix that plays a key role in cancer cell metastasis (5). In the case of HCC and the surrounding uninvolved liver tissue, the BM is primarily made up of three components: fibronectin (FN), laminin (LAM), and collagen IV (Coll IV) (6). BM is known to affect numerous physiological and pathological activities of cells including cell proliferation, adhesion, migration, and vascular remodeling (7, 8). As a result, in most cancers, BM plays a crucial role in driving cell metastasis (5, 9, 10). Due to the significant role of BM in cancer metastasis, it is an ideal target for anticancer drugs. Previous studies have found that stable markers can be created using different gene sets such as cuproptosis and necroptosis to predict the prognosis of HCC patients (11, 12). Recently, Jayadev et al. have redefined 222 BM-related genes (BMRG) and proteins (13), but a robust prognostic model based on BMRG in HCC is yet to be developed.

In this study, we screened 222 BMRG and identified 4 that were used to construct the basement membrane-related gene prognostic index (BMRGI). This index helps to more accurately predict the prognosis of patients with hepatocellular carcinoma (HCC). Furthermore, we evaluated the clinical relevance and impact of BMRGI on the tumor microenvironment. More importantly, we identified CTSA as a key BMRG in HCC, and comprehensively analyzed the expression differences of CTSA in HCC, and have confirmed that the expression of CTSA has a significant impact on the proliferation, migration, and metastasis of HCC cells.

## 2 Methods and materials

### 2.1 Data download and processing

The mRNA expression data of HCC patients with corresponding clinical information and somatic mutation data were downloaded from The Cancer Genome Atlas (TCGA, https://portal.gdc.cancer.gov/) database, and the mRNA expression data and clinical information from the Japan-HCC cohort were downloaded at International Cancer Genome Consortium (ICGC, https://dcc.icgc.org/). When performing correlation data analysis, we excluded cases with missing data. Finally, when performing prognostic analysis, we chose to exclude cases with a survival time ≤30. The single-cell RNA seq (scRNA-seq) data of 4 HCC patients were obtained from GSE146115 in the GEO database (https://www.ncbi.nlm.nih.gov/geo/), with a total of 27227 genes and 3200 cells obtained.

### 2.2 Screening of WGCNA and differential BMRG

The “WGCNA” package was used to construct the gene co-expression network of BMRG in the TCGA-HCC dataset (14). The core module was considered the one with the highest Pearson coefficient and also the one most associated with clinical traits. Furthermore, we analyzed differentially expressed genes (|log_2_FC| > 0.585, False Discovery Rate (FDR)< 0.05) in the TCGA-HCC dataset using the “Limma” package. Finally, we further investigated the 47 common genes.

### 2.3 Construction and verification of BMRGI

47 common genes were analyzed by univariate Cox analysis based on the “survival” package, and potential BMRG affecting the overall survival of HCC patients were screened out (p<0.05). Then, these candidate genes were analyzed by using the least absolute shrinkage and selection operator (LASSO). Based on the analysis results, we established a four-gene optimal prognostic model. The calculation formula of BMRGI for each HCC patient is as follows:


$$BMRGI=\sum_{i=1}^{n}Expression\left(i\right)*Coefficient\left(i\right)$$

Where X refers to the expression level of the selected gene, and Coef is the coefficient of the selected gene. In addition, the same calculation method is applied to the verification queue ICGC. According to the median BMRGI, HCC patients were divided into high BMRGI group and low BMRGI group. Kaplan–Meier curves were used to assess differences in OS between different BMRGI groups.

### 2.4 scRNA-seq data processing and analysis

The Seurat package is used for preprocessing and filtering of scRNA-seq (15). The PercentageFeatureSet function is used to calculate the mitochondrial gene content in cells. We further analyzed the cells in which the number of genes was >200 and the proportion of mitochondrial genes was<10%. We set the number of principal components (PC) to 20, the resolution to 0.4, and the 1500 genes with the largest variation between cells to cluster the cells.

### 2.5 CellChat analysis

We use CellChat to quantify and infer the communication links between different cell types from scRNA-seq data, and identify the signal input and output among them. In this study, we filtered for cell communication of less than 10 cells.

### 2.6 Gene ontology analysis, kyoto encyclopedia of genes and genomes analysis and gene set enrichment analysis

The “Limma” package was used to analyze differentially expressed genes (DEGs) between high and low BMRGI groups (|log_2_FC| > 1, FDR< 0.05). GO analysis was performed based on the “ clusterProfiler” package. KEGG analysis was performed based on the “clusterProfiler”, “org.Hs.eg.db”, “enrichplot” package. In addition, we also performed GSEA using the “clusterProfiler” package to explore biological differences among different BMRGI groups.

### 2.7 Analysis of immunological properties

The enrichment scores of 16 immune-related cells and 13 immune-related terms in HCC samples were calculated using the ssGSEA algorithm based on the R packages “GSVA” and “GSEABase”.

We also summarized common immune checkpoint molecules and HLA family genes, and analyzed the correlation between BMRGI and the expression of each gene, and displayed it with a radar map. Furthermore, we explored the somatic mutation profile of TCGA-HCC samples and listed the top10 mutation-prone genes in different BMRGI subgroups. In addition, we also compared the difference of TMB in the high BMRGI group and the low BMRGI group. Finally, the TIDE (http://tide.dfci.harvard.edu/) algorithm was used to predict and evaluate the response of HCC patients to immunotherapy.

### 2.8 Sensitivity analysis of common drugs

We use the R package “oncoPredict” (16) for the evaluation of common drug sensitivities.

### 2.9 Identification of core BMRG

SVM-REF (17), LASSO (18)and RandomForest (19) are commonly used machine learning methods with excellent classification performance. In biology-related research, it is often used for the screening of characteristic genes (20). In this study, we use these three types of machine learning to filter out characteristic BMRG, and use intersection to filter out the most critical BMRG.

### 2.10 Multilevel expression verification of CTSA

We analyzed the differential expression of CTSA at the mRNA level of HCC tissues online from the GEPIA2 database (http://gepia2.cancer-pku.cn/#index) (combined samples from TCGA and GTEx databases) (21). In addition, we analyzed the expression differences of CTSA at the protein level in the CPTAC database online using the UCLCAN database (http://ualcan.path.uab.edu/index.html) (22). Finally, the HPA database obtained the immunohistochemical images of CTSA in normal liver tissues and HCC tissues (23), and obtained the basic information of the corresponding tissue samples.

### 2.11 RNA extraction, and real-time quantitative PCR

Cell total RNA was extracted using Trizol reagent (Invitrogen, USA)). RNA extraction and RT-qPCR as previously described (24). Briefly, RNA was reversed to cDNA using PrimeScript™ RT Master Mix (Takara Bio, JAPAN). Fluorescence quantification was performed by TB-Green qPCR (Takara Bio, JAPAN) and normalized to β-actin. The information of all designed primers is listed in **Supplementary Table 1**.

### 2.12 Cell culture, transient transfection

All cell lines used in this study (including normal liver cell line LO2 and HCC cell lines HEPG2, BEL7402 and HCCLM3) were donated by Dr. Dai (25). All cell lines were cultured in complete DMEM medium (DMEM medium with 10% fetal bovine serum and 1% penicillin-streptomycin). Transient transfections were performed using jetPRIME Transfection Reagent (Polyplus, China) and followed the manufacturer’s instructions. siRNA sequences were designed by Tsingke Biotechnology Co., Ltd. The SiCTSA sequence is as follows, SiCTSA-1: sense-GCCUCUUUCCGGAGUACAA; antisense-UUGUACUCCGGAAAGAGGC. SiCTSA-2: sense-CUGCUUAGCUCACAGAAAU; antisense-AUUUCUGUGAGCUAAGCAG.

### 2.13 Cell counting kit-8 (CCK8) experiment

We planted 2×10^3^ cells in a 96-well plate, and set 5 replicate wells in each group, cultured them for 0 hour, 24 hours and 48 hours, respectively, and then added 10ul CCK8 reagent (Targetmol, USA) and incubated at 37°C for 2 hours. Absorbance was then measured at 450 nm using a microplate reader (TECAN, Switzerland).

### 2.14 Transwell experiment

We planted 5×10^4^ cells in the upper chamber (Corning, USA) containing 250ul serum-free medium. The upper chamber was without Matrigel (Corning, USA) for migration experiments, with Matrigel for invasion experiments, and the lower chamber add 800ul complete medium. After 24 hours of incubation, the cells were fixed with 4% paraformaldehyde and stained with 0.1% crystal violet. The cells on the upper surface of the upper chamber were wiped with a cotton swab, photographed under a microscope (Leica, Germany) at 100 times, and then counted.

### 2.15 Statistical analysis

All bioinformatics analyzes were performed on R software (version 4.1.2). Continuous variables that were not normally distributed were tested using the Wilcoxon test. Correlation analysis between BMRGI and drug IC50 was performed using the spearman method. The Kaplan-Meier method was used to draw the survival curves of different subgroups. All experimental data were analyzed for variance using Student’s T-test. p or FDR< 0.05 represents a statistical difference.

## 3 Result

### 3.1 WGCNA identified BM key module genes

**Figure 1:**
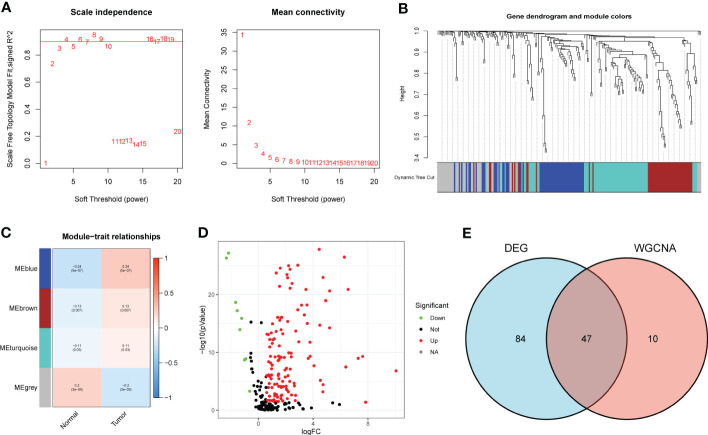
DEG screening of key module genes. **(A)** Scale independence and mean connectivity. **(B)** Gene clustering dendrogram, a total of 4 modules were identified. **(C)** Correlation heatmap of modules and clinical traits. **(D)** Volcano plot for BMRG differential analysis. **(E)** Venn diagram showing common genes of key module genes and differential genes.

According to the Materials and methods section, we identified 222 BMRG. First, we conducted WGCNA on 222 BMRG. By setting a minimum of 25 genes within a module, module connectivity (**Figure 1A**), 4 modules were finally identified (**Figure 1B**). According to the correlation thermograms of the modules, we found that the blue modules had the highest correlations with clinical traits (**Figure 1C**). Therefore, we choose the blue module for further analysis. Second, we performed differential analysis on 222 genes, and the results showed a total of 131 DEGs, of which 122 BMRG were up-regulated and 9 BMRG were down-regulated (**Figure 1D**). Furthermore, we showed the correlation of blue module genes with DEGs by Venn diagram, and finally obtained 47 common genes (**Figure 1E**).

### 3.2 Construction of BMRGI for HCC patients

**Figure 2:**
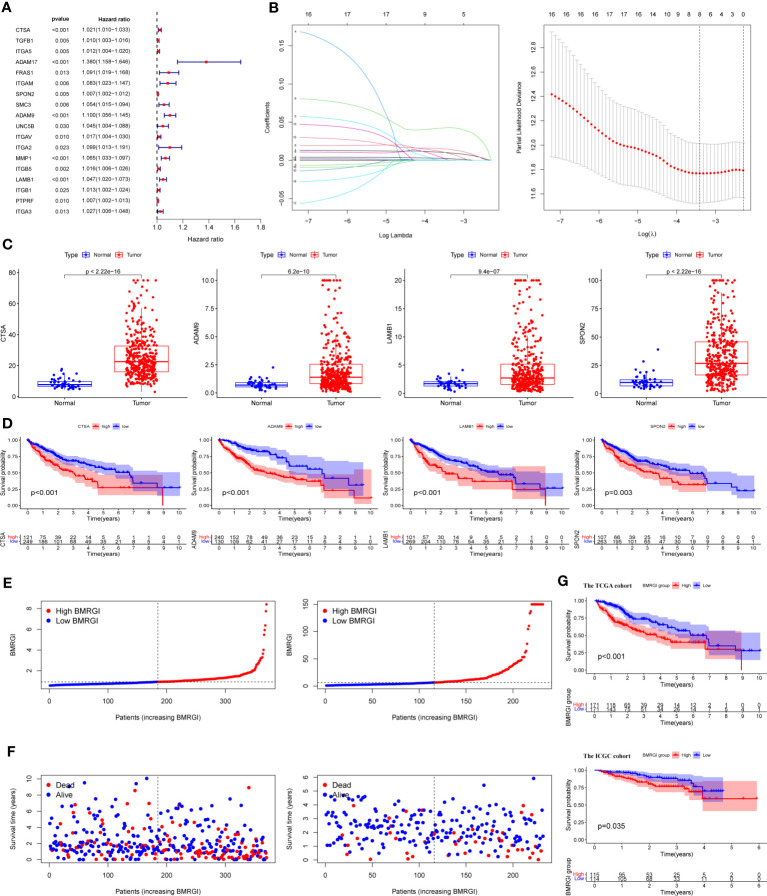
Construction of BMRGI and verification of its prognostic value. **(A)** Univariate Cox regression analysis of common genes. **(B)** LASSO analysis. **(C)** Expression difference analysis of CTSA, ADAM9, LAMB3 and SPON2. **(D)** Kaplan-Meier survival curves of CTSA, ADAM9, LAMB3 and SPON2. **(E)** Visual distribution of risk scores for TCGA cohort and ICGC cohort. **(F)** Survival status and time of TCGA and ICGC cohort. **(G)** Kaplan-Meier survival curves of BMRGI in TCGA and ICGC cohort.

We first performed univariate Cox analysis on 47 common genes, and the results showed that 18 BMRG were risk factors affecting OS in HCC patients (**Figure 2A**). LASSO analysis was further performed on 18 prognostic genes, and finally we identified 4 BMRG (**Figure 2B**). In addition, we also analyzed the expression differences and prognostic value of the 4 BMRG. Differential analysis showed that CTSA, ADAM9, LAMB3, and SPON2 were highly expressed in HCC (**Figure 2C**), and kaplan-meier analysis showed that high expression usually means poor prognosis (**Figure 2D**). Finally, we constructed the basement membrane-related gene prognostic index BMRGI based on the results of LASSO analysis.

According to the median BMRGI, HCC patients were divided into high BMRGI group and low BMRGI group. We use TCGA-HCC as the training cohort and ICGC as the validation cohort. First, we showed the risk scores of the training cohort and validation cohort more intuitively (**Figure 2E**). Furthermore, in both the TCGA-HCC cohort and the ICGC cohort, the higher the BMRGI, the shorter the survival time of HCC patients (**Figure 2F**). Finally, we performed a kaplan-meier analysis, and the results showed that the OS of the high BMRGI group was significantly lower than that of the low BMRGI group in both the TCGA cohort and the ICGC cohort (**Figure 2G**).

### 3.3 Single-cell transcriptional profiling and cell-cell interactions in HCC tissue

**Figure 3:**
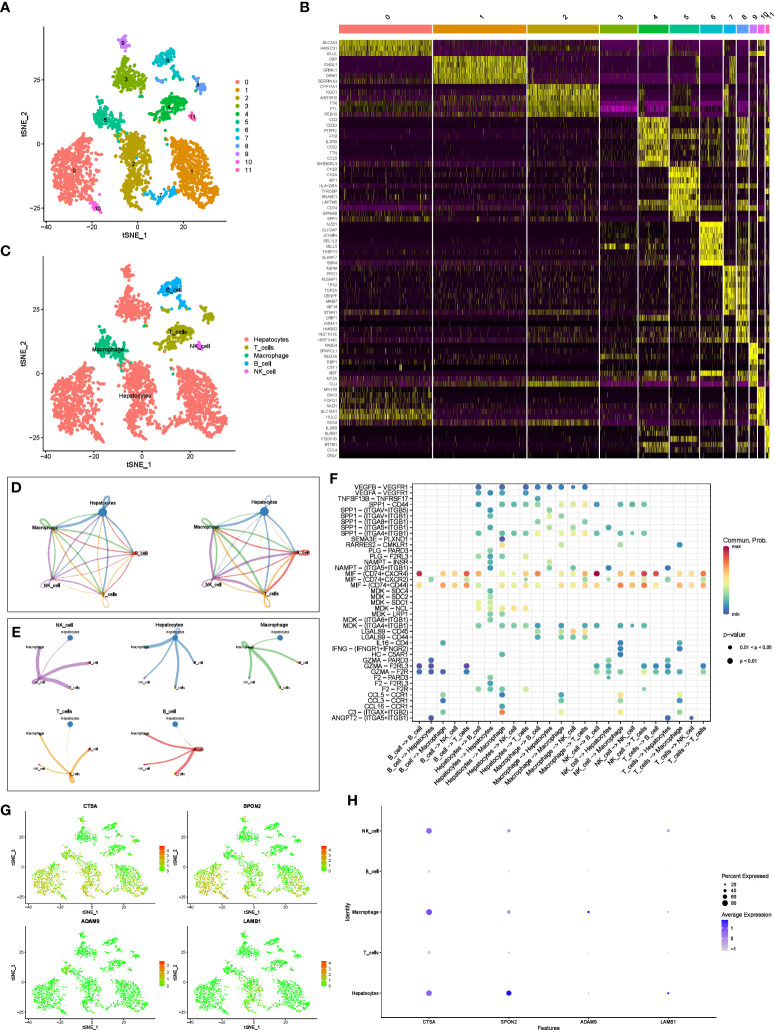
scRNA-seq analysis and CellChat Analysis **(A)** tSNE analysis to classify cell clusters. **(B)** Heatmap showing highly expressed genes in cell clusters. **(C)** The “SingleR” package annotates cell clusters into 5 cell types. **(D)** Network diagram of the number and weight of connections between different cell types. **(E)** Diagram of the communication network between cells and other cells. **(F)** Bubble diagram of receptor ligand molecules involved in cell communication. **(G, H)** Distribution and expression levels of model genes in 5 cell types.

We used tSNE to perform dimensionality reduction and clustering on the preprocessed scRNA-seq data, and finally obtained 12 clusters (**Figure 3A**). In addition, we also displayed the most significantly expressed genes in the 12 clusters using a heatmap (**Figure 3B**). Cell types were automatically annotated by the SingleR package, and these 12 clusters were clustered into 5 cell types, including Hepatocytes, T cells, Macrophage, B cell and NK cell (**Figure 3C**).

In addition, we further evaluated the interactions between different cells using the “CellChat” package. **Figure 3D** shows the number and weight of interactions among the five cell types. Furthermore, we present these results separately for a clearer picture of the strength of cell-cell interactions (**Figure 3E**). Overall, Hepatocytes rarely act as receptors for signals from the other four types of immune cells, but they can communicate with immune cells by emitting signals. Immune cells interact and receive signals frequently. In addition, receptor ligand molecules when mediating cell-cell interactions are shown in **Figure 3F**.

Finally, we explored the distribution of model genes in different cell types and showed the expression levels of the model in different cell types in bubble plots (**Figures 3G, H**). In brief, CTSA was highly expressed in hepatocytes, NK cells, and macrophages, SPON2 was highly expressed only in hepatocytes, whereas ADAM9 and LAMB1 were expressed at low levels in all cell types.

### 3.4 Comprehensive analysis of clinical parameters in HCC patients

**Figure 4:**
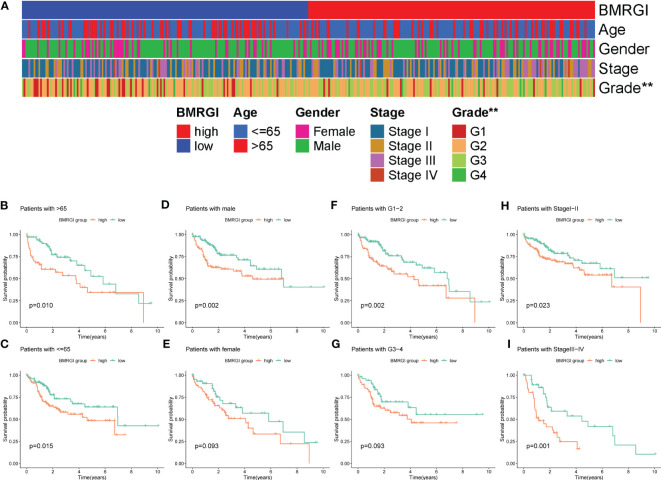
Correlation analysis between BMRGI and clinicopathological features. **(A)** Heatmap of the distribution of clinical case characteristics of patients in the high BMRGI group and low BMRGI group. **(B, C)** Kaplan-meier survival curves of high BMRGI group and low BMRGI group in different age groups. **(D, E)** Kaplan-meier survival curves of high BMRGI group and low BMRGI group in different gender groups. **(F, G)** Kaplan-meier survival curves of high BMRGI group and low BMRGI group under different grading groups. **(H, I)** Kaplan-meier survival curves of high BMRGI group and low BMRGI group in different stages.

As shown in **Figure 4A**, the heat map of BMRGI and common clinicopathological parameters, the results showed that the tumor stages of HCC patients with different BMRGI groups had statistical differences. In addition, we further determined the prognostic value of BMRGI in patients with different pathological features. The results showed that the high BMRGI group had significantly worse OS than the low BMRGI group in HCC patients with different clinicopathological parameters (age, gender, tumor grade and stage) (p< 0.1, **Figures 4B–I**). These results suggest that our BMRGI can effectively predict the prognosis of HCC patients with different clinicopathological features.

### 3.5 Construction and evaluation of clinical nomogram based on BMRGI

**Figure 5:**
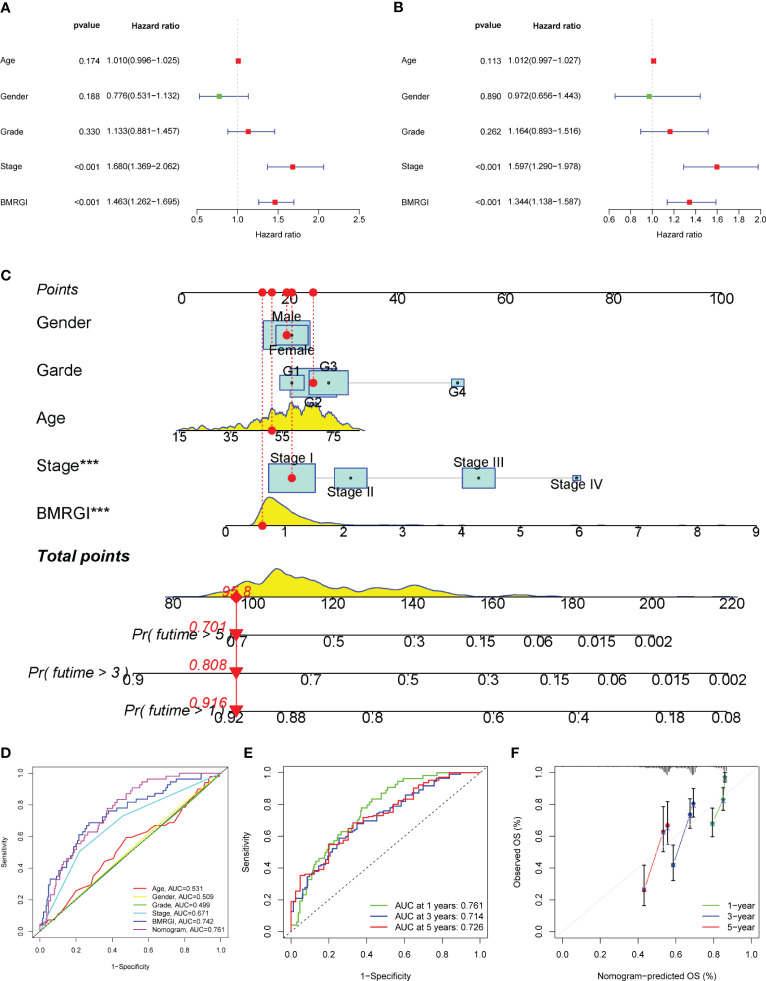
Construction of clinical nomogram. **(A, B)** Forest plots for univariate and multivariate Cox regression analysis. **(C)** Nomogram combining common clinical parameters and BMRGI. **(D)** ROC curves for clinical parameters, BMRGI and nomogram. **(E)** ROC curves of nomograms predicting 1-year, 3-year and 5-year survival rates. **(F)** Calibration curves for nomograms.

In order to construct a more practical and stable nomogram, we incorporated several common clinicopathological parameters (age, gender, tumor grade and stage). Univariate Cox analysis showed that tumor stage and BMRGI were risk factors affecting the prognosis of HCC patients (**Figure 5A**). Multivariate Cox analysis confirmed that tumor stage and BMRGI were independent risk factors affecting the prognosis of HCC patients after adjusting for other clinicopathological parameters (**Figure 5B**). Given the high correlation between BMRGI and prognosis of HCC patients. We constructed a new nomogram combining common clinicopathological parameters and BMRGI (**Figure 5C**). We first evaluated the AUC value of various indicators to predict the prognosis of HCC patients using the ROC curve, and the results showed that the ability of BMRGI to predict the prognosis of HCC patients was significantly better than other clinicopathological features (including the classic indicator tumor stage), and the nomogram constructed based on this further improved the accuracy of predicting the prognosis of HCC patients (**Figure 5D**). In addition, the excellent accuracy and robustness of the nomogram in assessing the 1-year, 3-year, and 5-year survival of patients was further illustrated by ROC curves and calibration curves (**Figures 5E, F**).

### 3.6 GO, KEGG and GSEA analysis related to BMRGI

**Figure 6:**
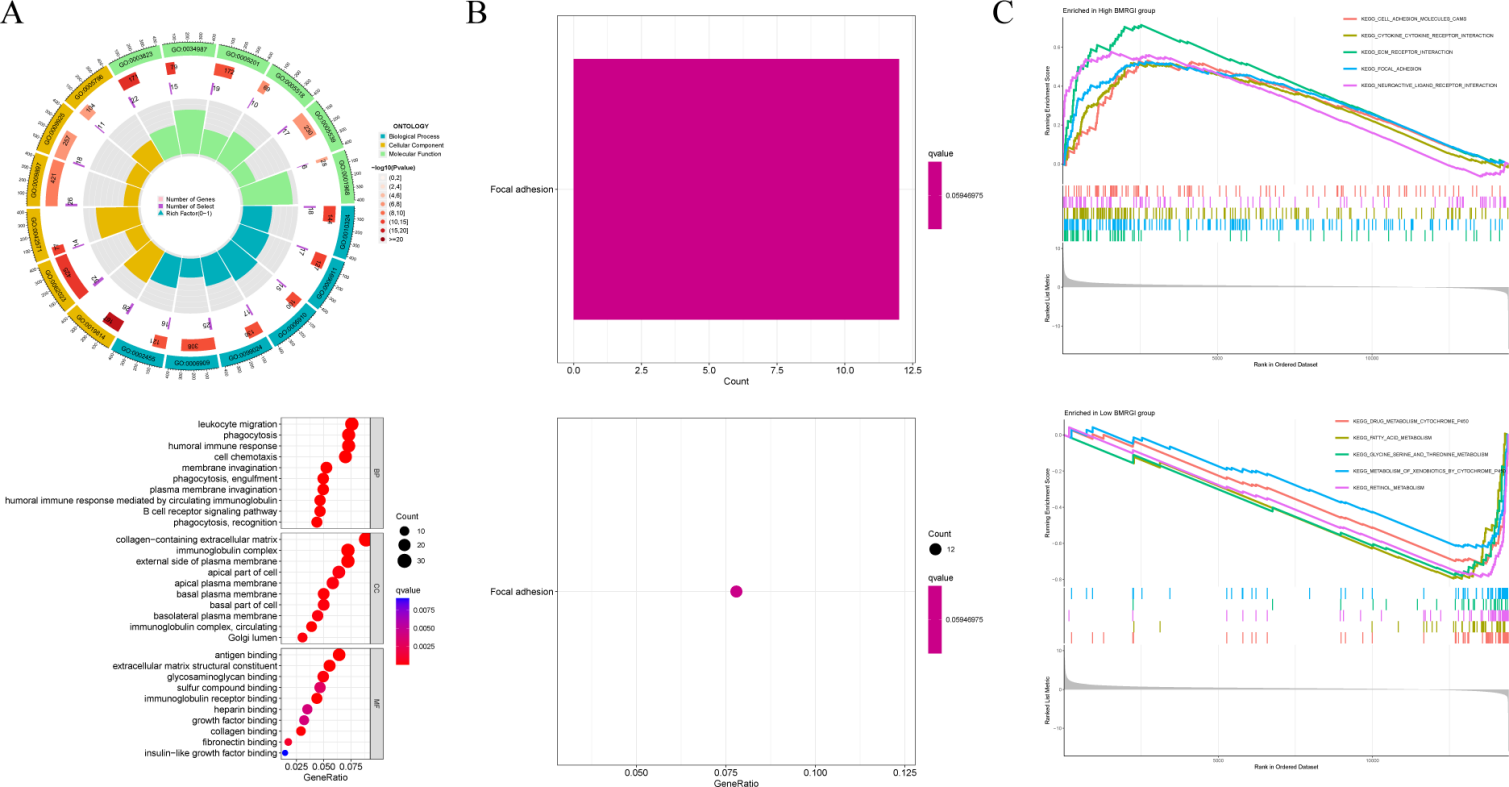
GO analysis, KEGG analysis and GSEA analysis. **(A)** Circle and bubble charts for GO analysis. **(B)** Barplot and bubble charts for KEGG analysis. **(C)** GSEA analysis of high BMRGI group and low BMRGI group.

First, we analyzed the genetic differences between the high BMRGI group and the low BMRGI group (|log_2_FC| > 1, FDR< 0.05). Based on these differential genes, we further performed GO analysis and KEGG analysis to explore their biological characteristics. GO analysis results showed that, in terms of biological process, DEGs were mainly enriched in “ membrane invagination, phagocytosis, engulfment, phagocytosis, recognition, plasma membrane invagination, phagocytosis, humoral immune response mediated by circulating immunoglobulin, humoral immune response, B cell receptor signaling pathway, cell chemotaxis, leukocyte migration”. In terms of cellular composition, DEGs were mainly enriched in “immunoglobulin complex, collagen−containing extracellular matrix, immunoglobulin complex, circulating, external side of plasma membrane, basal plasma membrane, Golgi lumen, basal part of cell, apical plasma membrane, basolateral plasma membrane, apical part of cell”. and in terms of molecular functions, DEGs were mainly enriched in “antigen binding, immunoglobulin receptor binding, extracellular matrix structural constituent, collagen binding, glycosaminoglycan binding, fibronectin binding, sulfur compound binding, heparin binding, growth factor binding, insulin−like growth factor binding” (**Figure 6A**). The results of KEGG analysis showed that DEGs were only enriched in the Focal adhesion signaling pathway (**Figure 6B**).

In addition, GSEA analysis was further carried out in this study. The results showed that the signal pathways affected by the high BMRGI group were mainly enriched in “ KEGG CELL ADHESION MOLECULES CAMS, KEGG CYTOKINE CYTOKINE RECEPTOR INTERACTION, KEGG ECM RECEPTOR INTERACTION, KEGG FOCAL ADHESION, KEGG NEUROACTIVE LIGAND RECEPTOR INTERACTION”. The signal pathways affected by the low BMRGI group were mainly enriched in “KEGG DRUG METABOLISM CYTOCHROME P450, KEGG FATTY ACID METABOLISM, KEGG GLYCINE SERINE AND THREONINE METABOLISM, KEGG METABOLISM OF XENOBIOTICS BY CYTOCHROME P450, KEGG RETINOL METABOLISM”(**Figure 6C**).

### 3.7 Comprehensive analysis of the correlation between BMRGI and tumor microenvironment

**Figure 7:**
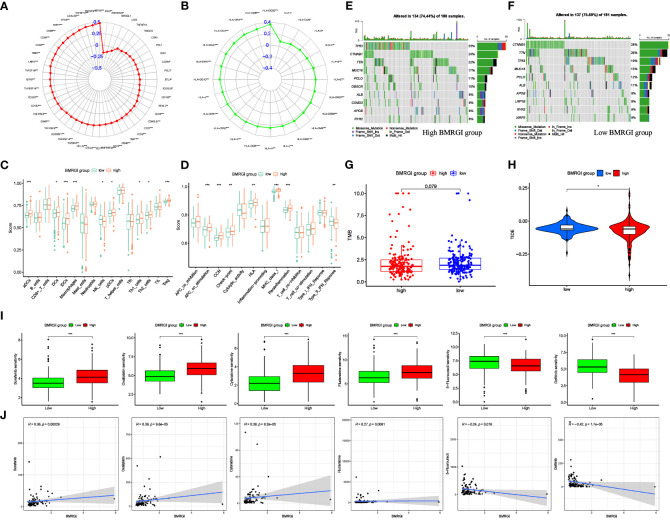
Correlation analysis between BMRGI and tumor microenvironment and common drug sensitivity. **(A)** Correlation between BMRGI and immune checkpoint molecules. **(B)** Correlation between BMRGI and HLA family molecules. **(C)** Differences in immune cell infiltration between high and low BMRGI groups. **(D)** Differences in immune-related terms between high and low BMRGI groups. **(E)** TOP10 mutated genes in high BMRGI group. **(F)** TOP10 mutated genes in low BMRGI group. **(G)** Difference analysis of TMB between high BMRGI group and low BMRGI group. **(H)** Difference analysis of TIDE scores between high BMRGI group and low BMRGI group. **(I)** Difference analysis of IC50 values of 6 commonly used drugs in high BMRGI group and low BMRGI group. **(J)** Correlation analysis between IC50 values of 6 commonly used drugs and BMRGI. * represents p < 0.05, ** represents p < 0.01, and *** represents p < 0.001.

In view of the important guiding significance of immune checkpoint molecules and HLA family molecules in immunotherapy. We analyzed the correlation between BMRGI and 48 common immune checkpoint molecules and 24 HLA family molecules. The results showed that BMRGI was significantly positively correlated with 41 immune checkpoint molecules as well as 23 HLA family molecules (**Figures 7A, B**). In addition, we assessed the levels of 16 immune-related cells and 13 immune-related terms in tissue samples from HCC patients using ssGSEA. In terms of immune-related cells: Compared with the low BMRGI group, aDCs, DCs, iDCs, Macrophages, pDCs, Th1_cells, Th2_cells, and Treg were significantly increased in the high BMRGI group, while NK_cells were significantly decreased (**Figure 7C**). In terms of immune-related terms, compared with the low BMRGI group, the levels of APC_co_Stimulation, CCR, Check-point, HLA, MHC_class_I, and Parainflammation were significantly increased, while Type_II_IFN_Reponse was significantly decreased (**Figure 7D**). Then, we analyzed the somatic mutation profile of HCC patients and identified the top 10 mutated genes in the high and low BMRGI groups. The results showed that TP53 mutations were significantly lower in the high BMRGI group than in the BMRGI group (**Figures 7E, F**). However, there was no statistical difference in TMB between the high and low BMRGI groups (**Figure 7G**). Finally, we assessed the sensitivity to immunotherapy in the high and low BMRGI groups. The results showed that TIDE was lower in the high BMRGI group, indicating that the lower the possibility of immune escape, the better the effect of immunotherapy (**Figure 7H**).

### 3.8 Screening for sensitive drugs in HCC patients

We evaluated and observed the differences in sensitivity to 6 common drugs in HCC patients between the two groups. The lower the IC50 value, the higher the sensitivity to the drug. The results showed that patients in the low BMRGI group were more sensitive to sorafenib, oxaliplatin, cytarabine and fludarabine, whereas patients in the high BMRGI group were more sensitive to 5-fluorouracil and gefitinib higher (**Figures 7I, J**). All in all, these results provide a good reference for clinical medication.

### 3.9 Identification of key BMRG

**Figure 8:**
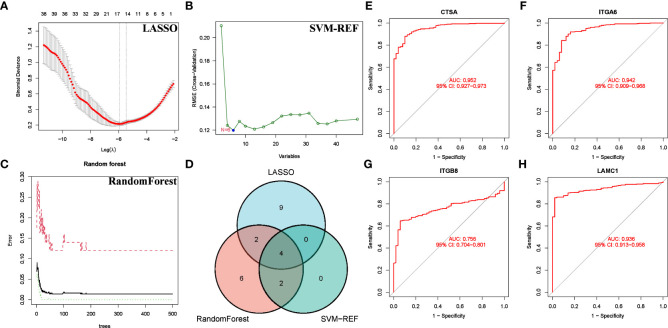
Screening for Feature BMRG. Characteristic genes in DEGs selected by LASSO **(A)**, SVM-SEF **(B)** and RandomForst **(C)**. **(D)** The Venn diagram shows the common genes of the three algorithms. **(E–H)** ROC curve of common genes.

We conducted a more refined analysis based on the 47 common genes screened above. First, we identified marker molecules of HCC by 3 machine learning methods (LASSO, SVM-REF, and RandomForest) (**Figures 8A–C**). Among them, CSTA, ITGA6, ITGB8 and LAMC1 are common marker molecules (**Figure 8D**). Then we evaluated the diagnostic value of the four marker molecules through the ROC curve, and the results showed that CSTA (AUC = 0.952), ITGA6(AUC = 0.942), ITGB8(AUC = 0.756) and LAMC1(AUC = 0.936) all had high diagnostic value (**Figures 8E–H**). At the same time, we found that CTSA not only has the highest diagnostic value, but also constitutes one of the members of BMRPI. Therefore, we considered CTSA as the most critical BMRG in HCC for further study.

### 3.10 Multilevel expression verification and *in vitro* functional exploration of CTSA

**Figure 9:**
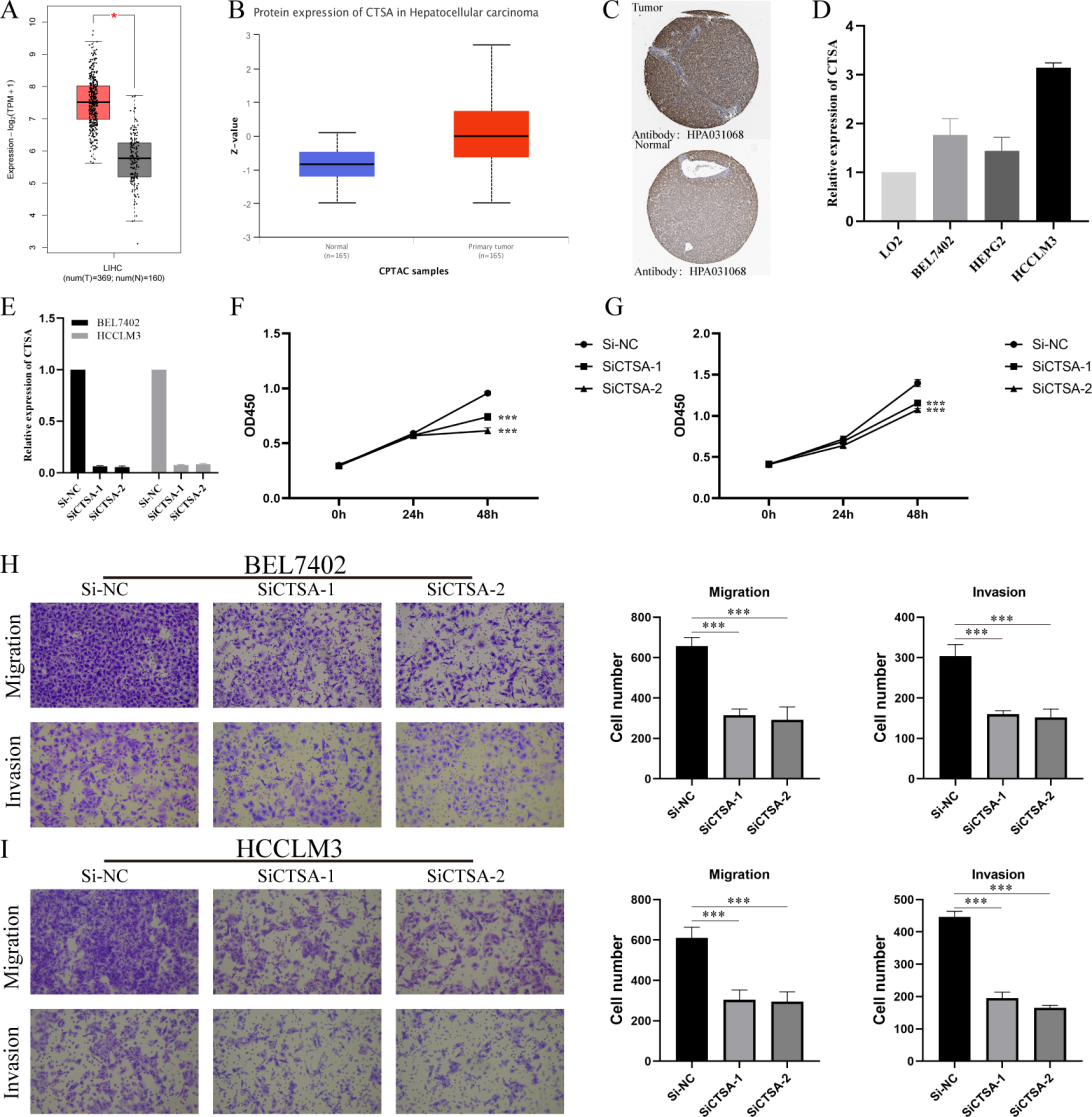
Multidimensional expression validation of CTSA and modulation of HCC oncogenic capacity *in vitro*. **(A)** Differential analysis of mRNA expression of CTSA in HCC in GEPIA2.0 database. **(B)** Differential analysis of protein expression levels of CTSA in HCC from UALCAN database. **(C)** IHC images of CTSA in HCC tissues and normal liver tissues from HPA database. **(D)** Expression levels of CTSA in normal liver cell lines and HCC cell lines. **(E)** Knockdown efficiency of siCTSA in BEL7402 and HCCLM3 cells. **(F, G)** CCK8 assay detects the effect of knocking down CTSA on the proliferation ability of BEL-7402 (Left) and HCCLM3 (Right). **(H)** Transwell assay was used to detect the effect of knocking down CTSA on the migration and invasion of BEL7402 cells. **(I)** Transwell assay was used to detect the effect of knocking down CTSA on the migration and invasion of HCCLM3 cells. *** represents p < 0.001.

We further investigated the differential expression of CTSA in HCC. First, we searched through GEPIA2.0, and the results showed that CTSA was highly expressed in HCC (**Figure 9A**). Second, we explored the differential expression of CTSA at the protein level. The UALCAN database (https://ualcan.path.uab.edu/index.html) showed that the protein level of CTSA in HCC was significantly higher than that in the normal group (**Figure 9B**). Likewise, the HPA database showed that CTSA was highly expressed in HCC tissues compared with normal liver tissues (**Figure 9C**). Finally, we detected the expression of CTSA in normal liver cell lines (LO2) and liver cancer cell lines (BEL7402, HEPG2, HCCLM3), and the results showed that the expression level of CTSA in liver cancer cell lines was significantly higher than that in normal liver cell lines (**Figure 9D**).

To gain insight into the *in vitro* function of CTSA in HCC, we characterized the oncogenic phenotype of HCCLM3 and BEL-7402 cells (SiCTSA-1 and SiCTSA-2) with CTSA knockdown. The RT-qPCR results showed that siCTSA-1 and siCTSA-2 could significantly inhibit the CTSA expression of HCC cells (BEL-7402 and HCCLM3 cells) (**Figure 9E**). We studied the role of CTSA in HCC cell proliferation by CCK8 assay, and the role of CTSA in HCC cell migration and invasion using Transwell assay. CCK8 assay and Transwell assay analysis showed that the reduction of CTSA impaired the proliferation (**Figures 9F, G**), migration (**Figure 9H**) and invasion (**Figure 9I**) abilities of HCC cells (BEL7402 and HCCLM3).

## 4 Discussion

HCC is the most prevalent histological type of primary liver cancer, known for its high metastatic and recurrence characteristics (4). Unfortunately, most HCC patients are diagnosed at an advanced stage, which significantly reduces the chance of curative treatment and leads to a poor prognosis (26). BM, as an important component of the extracellular matrix, is an important barrier that cancer cells must overcome to form metastasis (5, 27). Numerous studies have demonstrated the association between the main components of BM and HCC tumor metastasis, as well as poor prognosis (28–30). Current study shows that systematic analysis of specific gene sets achieves promising results in predicting cancer prognosis (31, 32). Despite advancements in research, there is still a lack of reliable prognostic models for HCC based on basement membrane genes. To address this gap, our study utilized WGCNA and machine learning to develop a strong prognostic index based on BMRG. Our model has demonstrated high accuracy in predicting the prognosis of HCC patients. This study utilized the TCGA-HCC dataset to identify 4 BMRG (CTSA, ADAM9, LAMB1, and SPON2) through WGCNA and machine learning techniques. These BMRG were used to construct BMRGI. Previous research has shown that all four BMRG are closely associated with HCC. Wang et al. discovered that CTSA has potential as a diagnostic and prognostic marker for HCC patients (33). In HCC, ADAM9 is known to be overexpressed and is responsible for inducing ROS generation, which in turn promotes HCC cell invasion (34). Additionally, LAMB1 has been shown to be regulated by the RNA helicase DDX24, which contributes to the malignant progression of HCC (35). However, the role of SPON2 in HCC is still a matter of debate. While high expression of SPON2 has been linked to poor prognosis in HCC patients (36), it has also been found to inhibit tumor metastasis by promoting the infiltration of M1-like macrophages (37). Taken together, these studies showed that the four BMRG were closely related to HCC and its prognosis, which indicated the correctness of our BMRGI based on them.

By analyzing the single-cell atlas of HCC tissue, we identified five types of cells present. Our findings suggest that hepatocytes are capable of acting as ligands to send signals to immune cells, while immune cells exhibit a weaker ability to send signals to liver cells. We conducted an analysis of the expression of model genes across various cell types and found that CTSA expression was particularly high in hepatocytes, NK cells, and macrophages. This suggests that any abnormal expression of CTSA could potentially impact the progression of HCC by influencing the immune microenvironment of HCC.

We conducted related validation on BMRGI. Survival analysis revealed that the prognosis of the high BMRGI group was significantly worse than that of the lower BMRGI group in the TCGA-HCC cohort. Furthermore, we validated the ability of BMRGI to predict the prognosis of HCC patients in the ICGC cohort. The study found that BMRGI is an independent risk factor for the prognosis of HCC patients, as determined by the results of multivariate Cox analysis. Additionally, ROC curve analysis revealed that BMRGI is a better predictor of HCC patient prognosis compared to other clinicopathological parameters. Subgroup analysis based on clinical characteristics demonstrated that BMRGI has a strong ability to predict prognosis for HCC patients with varying clinical characteristics. In order to facilitate clinical application and improve the accuracy of predicting the prognosis of HCC patients, we combined common clinicopathological parameters with BMRGI to construct a nomogram.

We conducted further analysis of the differentially expressed genes (DEGs) between the high and low BMRGI groups to investigate the biological properties of these subgroups. Our analysis, which included Gene Ontology (GO), Kyoto Encyclopedia of Genes and Genomes (KEGG), and Gene Set Enrichment Analysis (GSEA), revealed significant differences in biological processes related to immunity and cell adhesion (BM is closely related) between the different BMRGI subgroups.

This study demonstrates the accuracy of BMGPI construction (closely related to BM-related biological characteristics). Additionally, BMGPI effectively recognizes differences in the tumor immune microenvironment. Further analysis was conducted to determine the correlation between BMRGI and the tumor immune microenvironment. Previous research has shown that immune checkpoint molecules and HLA family molecules are strong predictors of response to immunotherapy (38–41). Therefore, we analyzed the correlation between BMRPI and immune checkpoint molecules and HLA family molecules. The results showed that BMRGI was positively correlated with most immune checkpoint molecules and HLA molecules, suggesting that BMRGI may also be a good biomarker for predicting immunotherapy response. Second, our results showed significant differences in terms of immune-related cells between high and low BMRGI groups, implying that different BMRGI subgroups may differ in response to immunotherapy. Interestingly, the mutation rate of TP53 in the high BMRGI group was significantly higher than that in the low BMRGI group, which may be one of the reasons for the poor prognosis in the high BMRGI group (42). However, overall TMB levels were not statistically different between the two groups. In this study, we used the TIDE algorithm to analyze the response to immunotherapy in various BMRGI subgroups of HCC patients. Our findings indicate that patients in the high BMRGI group had a lower TIDE score, suggesting that they may be less prone to immune escape and therefore have a better response to immunotherapy. Additionally, we evaluated the sensitivity of different BMRGI subgroups to six commonly used therapeutic drugs, providing valuable insights for clinical decision-making.

Finally, we screened 47 common gene by multiple machine learning methods and finally identified 4 BMRG: CTSA, ITGA6, ITGA8, and LAMC1. ROC analysis showed that these genes have high diagnostic value for distinguishing HCC. We found that CTSA not only had the highest diagnostic value (AUC:0.952), but also constituted one of the core members of BMRGI. Therefore, we regard CTSA as the most critical member of BMRG and conduct in-depth research. We verified that the expression of CTSA in HCC was significantly higher than that in normal tissues at the mRNA level and protein level by GEPIA2.0 database, UALCAN database and HPA database. In addition, we also verified by RT-qPCR that the expression of CTSA in HCC cell lines was significantly higher than that in normal liver cell lines. Since the oncogenic role of CTSA in HCC is still unclear, this prompted us to further explore the role of CTSA in HCC progression. More importantly, our *in vitro* cell experiments showed that the proliferation, migration and invasion abilities of HCC cell lines (BEL7402 and HCCLM3) were significantly reduced after CTSA knockdown.

Like other studies, even this study has some limitations and shortcomings. First, when we validated the prognostic value of BMRGI, we did not validate it in real cohorts. Second, the carcinogenesis of CTSA has not been explored by *in vivo* experiments. Finally, the specific molecular mechanism by which CTSA affects the progression of HCC was not elucidated in this study.

In conclusion, our study trained and validated a BMRGI that could effectively predict the prognosis of HCC patients based on 222 BMRG. Based on this, we also developed a nomogram for clinical application. The biological and immunological characteristics of BMRGI in HCC were explored through a series of bioinformatics methods, and some insights were provided for clinical immunotherapy and targeted therapy. Finally, we also verified the role of the key BMRG CTSA in HCC progression through *in vivo* functional experiments.

## Data availability statement

The original contributions presented in the study are included in the article/**Supplementary Material**. Further inquiries can be directed to the corresponding authors.

## Author contributions

PS and YY have constructed and devised the research. WS and JW performed data analysis and wrote the manuscript. ZW, MX and QL analyzed the data. WS performed the experiments. All authors contributed to the article and approved the submitted version.
